# Predicting Residual 21‐Hydroxylase Enzymatic Activity in Pediatric and Adult Congenital Adrenal Hyperplasia Patients: Towards Individualized Therapy

**DOI:** 10.1002/psp4.70086

**Published:** 2025-07-27

**Authors:** Davide Bindellini, Robin Michelet, Yersultan Mirasbekov, Qizong Lao, Charles Sukin, Wilhelm Huisinga, Deborah P. Merke, Charlotte Kloft

**Affiliations:** ^1^ Department of Clinical Pharmacy and Biochemistry Institute of Pharmacy, Freie Universitaet Berlin Berlin Germany; ^2^ Graduate Research Training Program PharMetrX Berlin Germany; ^3^ National Institutes of Health Clinical Center Bethesda Maryland USA; ^4^ Institute of Mathematics University of Potsdam Potsdam Germany; ^5^ Eunice Kennedy Shriver National Institute of Child Health and Human Development Bethesda Maryland USA

**Keywords:** 21‐hydroxylase deficiency, ACTH, congenital adrenal hyperplasia, cortisol, hydrocortisone, therapy individualization

## Abstract

Congenital adrenal hyperplasia (CAH) is a genetic disorder characterized by impaired cortisol production and consequent elevated adrenocorticotropic hormone (ACTH): CAH patients often require lifelong hydrocortisone therapy. Disease severity reflects residual 21‐hydroxylase enzyme activity, crucial for cortisol synthesis. Accurate assessment of residual enzymatic activity is key to developing individualized dosing. This study aimed to estimate enzymatic activity using a previously developed healthy adult ACTH‐cortisol model and to evaluate the potential for individualized therapy. Leveraging ACTH (*n* = 62) and cortisol (*n* = 66) concentrations from 51 (20 pediatric, 31 adult) untreated CAH patients, and assuming maximal cortisol production (E_max_) = 100% in healthy individuals, residual enzymatic activity was estimated as an E_max_ scaling factor. To assess proof‐of‐concept feasibility of individualized therapy, simulations of individual untreated 24‐h ACTH and cortisol profiles were performed, and for one patient hydrocortisone dosing regimens (15–25 mg/day in 3 doses, q4h or q6h) were compared to simulated untreated and healthy profiles. The original model failed to capture elevated ACTH in severe CAH and was refined to predict observed data across all patients. Using the refined model, estimated enzymatic activity was higher than in vitro values for adults, while children under 13 years old showed 31.6% of adult enzymatic activity. Shortening dosing intervals had a greater impact on reducing the patient's ACTH overexposure than increasing the daily dose. This model‐based approach captured in vivo endogenous cortisol production and enabled simulation‐based evaluation of individualized therapy in adults. In children, further validation of the ACTH‐cortisol dynamics model and enzymatic activity estimates is needed to evaluate individualized therapy.

**Summary**

- What is the current knowledge on the topic?
  ○ CAH is characterized by impaired cortisol production due to reduced 21‐hydroxylase enzymatic activity, requiring lifelong cortisol replacement therapy.
  ○ Residual enzymatic activity determines CAH severity, is associated with genotype, and is typically quantified based on in vitro studies.
- What question did this study address?
  ○ Could a model‐based approach be leveraged to estimate residual enzymatic activity in untreated pediatric and adult CAH patients and further be used to evaluate individualized dosing regimens?
- What does this study add to our knowledge?
  ○ Model‐based estimated enzymatic activity was higher than in vitro derived values, better capturing CAH phenotype. Children younger than 13 had lower enzymatic activity than adults.
  ○ The estimation of individual residual enzymatic activity allowed for evaluating dosing regimens for individual patients.
- How might this change drug discovery, development, and/or therapeutics?
  ○ This novel model‐based approach uses patient hormone levels to estimate residual enzymatic activity and offers an opportunity to individualize therapy beyond traditional genotype‐based in vitro studies.
  ○ Our findings support modeling patient hormonal data as a promising new method to individualize cortisol replacement therapy.

## 1 Introduction

Congenital adrenal hyperplasia (CAH) is a rare (1:10000–1:20000 in severe forms and 1:200–1:1000 in mild forms) [1, 2, 3] recessive genetic disease often requiring lifelong therapy and characterized by impaired cortisol production caused by, in approximately 95% of cases, *CYP21A2* mutations leading to defects in the 21‐hydroxylase enzyme [4, 5] (Figure S1). Cortisol production follows a circadian rhythm and is regulated by the hypothalamic–pituitary–adrenal (HPA) axis: Corticotropin releasing hormone (CRH) is secreted by the hypothalamus stimulating the secretion of adrenocorticotropic hormone (ACTH) by the pituitary gland, which then initiates cortisol production in the adrenal glands. Homeostasis is maintained by cortisol‐driven feedback inhibition of both CRH and ACTH release [6, 7, 8]. Hence, in CAH, the reduced enzymatic activity causes reduced cortisol production, which in turn results in alterations of the hormonal homeostasis due to the lack of cortisol‐driven feedback inhibition. Specifically, patients with CAH present with increased ACTH and ACTH‐driven androgen levels (Figure S1), resulting in virilization of females, premature pseudo puberty, and short stature in children, as well as infertility in both men and women [4, 5]. Additionally, aldosterone production, which also depends on the 21‐hydroxylase enzyme, may be insufficient and can result in potentially fatal electrolyte imbalances.

The severity of CAH is thus related to the degree of residual enzymatic activity. Therefore, clinical manifestations of CAH are typically grouped into three main categories based on the percentage of residual enzymatic activity: Salt wasting (SW, 0%–1%), simple virilizing (SV, 1%–5%) and non‐classic (NC, 20%–70%) [5, 9, 10, 11, 12]. However, in recent years, phenotypic manifestations of CAH have been considered as a continuum instead of discrete categories [5]. More than 200 different allelic variants have been identified [13, 14] and result in different degrees of residual 21‐hydroxylase enzymatic activity, and thus varied genotype‐related clinical manifestations of CAH. As CAH is a recessive disorder, in the case of heterozygote individuals, the clinical phenotypes of CAH were found to strongly correlate with the least affected allele [10, 12, 15, 16, 17, 18]. While generally there is a good genotype–phenotype correlation in CAH, in many cases, the distinctions between SW and SV, or SV and NC, are not as clear, highlighting the need for a more granular classification.

For CAH patients, cortisol replacement therapy with oral hydrocortisone (synthetic cortisol) is the most commonly adopted therapeutic strategy, aiming to mimic cortisol's physiological circadian rhythm and thus reducing ACTH and androgen overproduction [1, 5]. Currently, for SW and SV patients, the standard dosing regimen of immediate‐release hydrocortisone is 10–15 mg/m^2^/day divided into 3–4 doses (irregular dosing intervals) for pediatric patients and 15–25 mg/day divided into 2–3 doses (irregular dosing intervals) for adult patients [1, 5]. In contrast, no guidelines are available for NC patients, and decisions on treatment are based on clinical evaluation: As such, asymptomatic NC patients are usually untreated. As CAH is a genetic disorder, SW and SV patients require cortisol replacement therapy from birth, highlighting the need for therapy optimization and individualization in the pediatric population. Yet, given the scarcity of data from the pediatric population, attempts to extrapolate findings from adults to pediatrics offer an opportunity to improve cortisol replacement therapy for both populations.

When designing cortisol replacement therapy dosing regimens, the extent of endogenous cortisol production of an individual CAH patient should be taken into account to avoid under‐ or over‐exposure: the latter potentially leading to iatrogenic Cushing syndrome, with metabolic and cardiovascular adverse reactions, as well as decreased bone density and impaired growth and reproductive function [1, 5]. As the extent of endogenous cortisol production depends on the degree of residual enzymatic activity, retrieving such information would be of great importance for individualizing cortisol replacement therapy [19]. Typically, residual enzymatic activity yielding from specific pathogenic variants is quantified via in vitro or structure/activity in silico methodologies [20, 21, 22, 23, 24]. In this context, nonlinear mixed‐effects (NLME) modeling represents a powerful methodology for estimating parameters on the individual level, such as a patient's enzymatic activity. To enable this, we previously developed an NLME model based on data from healthy adults in which ACTH and cortisol dynamics were characterized and quantified [25]. Here, the aim was to extrapolate this model from healthy adults to adult patients and lastly to pediatric patients with CAH.

The aim of this study was to evaluate the use of a healthy adult model for endogenous ACTH and cortisol dynamics to estimate residual enzymatic activity in untreated pediatric and adult patients with CAH, with a focus on assessing potential differences between pediatrics and adults. Ultimately, by leveraging individual patient parameters, this study aimed to evaluate the potential of dose individualization in cortisol replacement therapy in the future.

## 2 Patients and Methods

**FIGURE 1:**
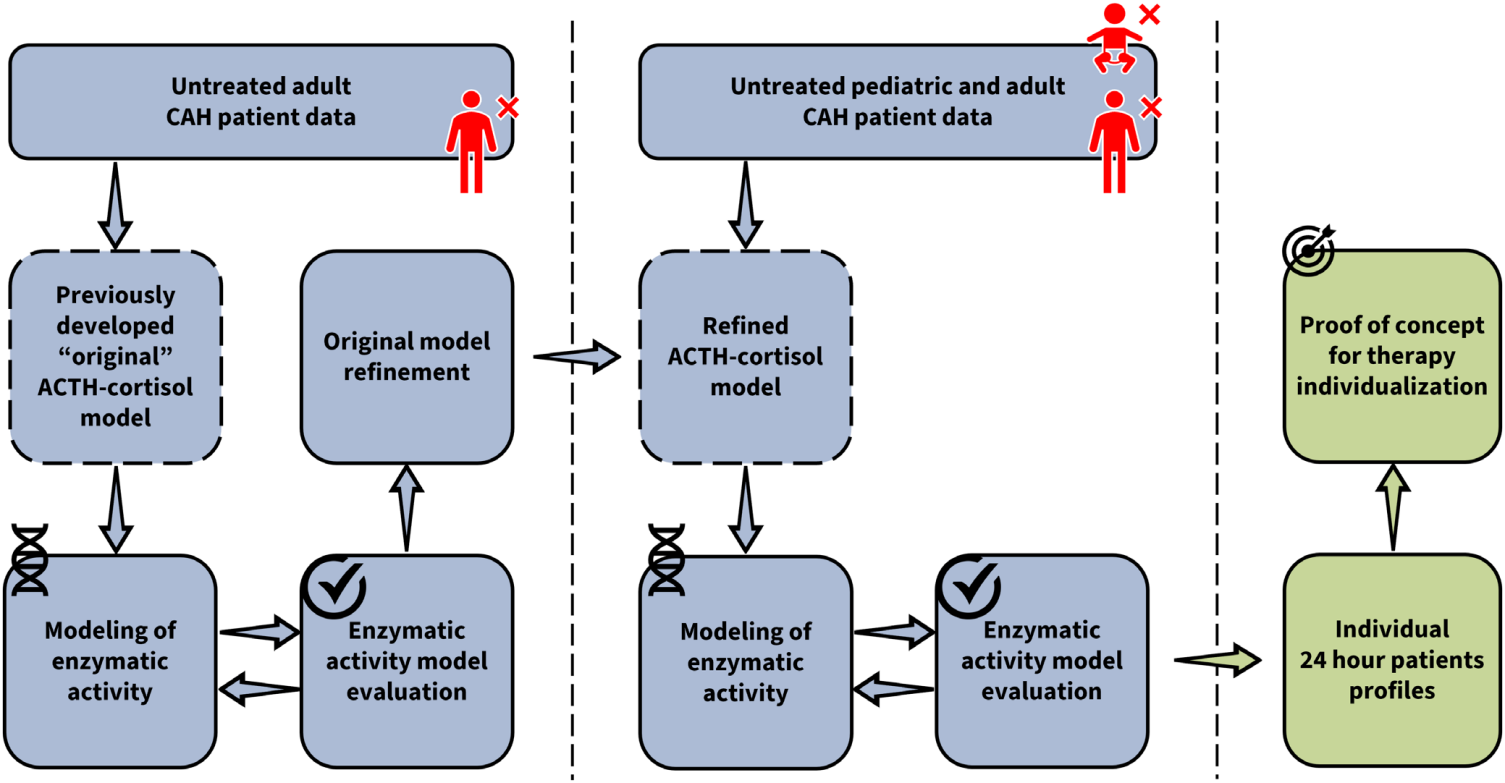
Schematic overview of the data, models and methodological workflow. Blue boxes with solid outline: Data/modeling steps, blue boxes with dashed outline: Underlying ACTH‐cortisol model, green boxes: Simulation steps. ACTH, adrenocorticotropic hormone; CAH, congenital adrenal hyperplasia.

A schematic overview of the data, models, and methodological workflow presented in the following sections is provided in Figure 1.

### 2.1 Untreated CAH Patient Data

Data from pediatric and adult untreated CAH patients was collected as part of the Natural History Study of Patients with Excess Androgen [26] (NCT00250159): Patients with known, either classic (SW or SV) or NC CAH between 0 and 99 years old were eligible. Patients underwent clinical and hormonal evaluations to establish their CAH phenotype, as well as DNA testing to identify their genotype and thereof the pathogenic variants responsible for the disease. Additionally, as part of hormonal analysis, ACTH and cortisol samples were collected and analyzed using either LC–MS/MS or chemiluminescence immunoassay (CLIA) (NIH, Bethesda, Maryland, USA). The study was approved by the Eunice Kennedy Shriver National Institute of Child Health and Development Institutional Review Board, and all patients (adults) or parents (children) provided written informed consent. Children at least 8 years old provided written assent.

### 2.2 Previously Developed “Original” ACTH‐Cortisol Model

A quantitative framework integrating endogenous ACTH and cortisol system dynamics and immediate‐release hydrocortisone pharmacokinetics was previously developed leveraging healthy adult data and is described in detail elsewhere [25]. A total of 325 endogenous cortisol concentrations, 310 endogenous ACTH concentrations, and 1865 cortisol concentrations following dexamethasone/hydrocortisone administration were analyzed. In brief, ACTH secretion was modeled using two surge functions to account for circadian rhythmicity (early morning peak time = 06:18, second peak time = 11:48). Cortisol production was exclusively dependent on ACTH concentrations and was modeled using a sigmoidal E_max_ model (E_max_ = 5400 nmol/h, EC_50,ACTH_ = 6.63 pmol/L, Hill factor = 2.94). Additionally, cortisol‐driven suppression of ACTH secretion was characterized using a sigmoidal I_max_ model (I_max_ = 99.9% fixed, IC_50,unbound cortisol_ = 4.60 nmol/L, Hill factor = 5.33). Cortisol and hydrocortisone disposition kinetics were assumed to be identical and were characterized by a two‐compartment model that included plasma protein binding to corticosteroid binding globulin (saturable) and albumin (linear) [25, 27, 28]. Lastly, immediate‐release hydrocortisone absorption was modeled using a transit compartment model. In this manuscript, the previously developed framework will be referred to as the “original model” (Equations S1–S10).

### 2.3 Modeling of Enzymatic Activity

To estimate 21‐hydroxylase residual enzymatic activity of CAH patients, all other model parameters from the original model were fixed. Enzymatic activity was assumed to be represented by E_max_, the maximal cortisol production rate, and healthy individuals were assumed to have 100% activity. Thus, enzymatic activity for CAH patients was estimated as an E_max_ scaling factor between 0 and 1 (0% and 100%). To ensure each individual enzymatic activity estimate to be between 0 and 1, parameters were estimated in the logit domain.

As differences between pediatric and adult CAH patients could be expected, only untreated adult patient data were initially analyzed. The estimation of typical enzymatic activity values was performed using a genotype‐based approach: A typical value was estimated per variant (least affected allele) and the inclusion of interindividual variability was tested for each variant separately. Once a best model was chosen, the need for refining the original model was evaluated, and the procedure is described in a following section.

For the parameters estimated with low precision and associated with low identifiability, log‐likelihood profiling (LLP) was performed and parameter estimates were fixed to the parameter values resulting in the minimum objective function value (OFV). To evaluate the identifiability and feasibility of precisely estimating enzymatic activity parameters, a model sensitivity analysis for E_max_ was performed by using deterministic simulations of ACTH and cortisol profiles with varying E_max_ values in steps of 10% between 0% (no activity) and 100% (healthy). Small differences in simulated ACTH and cortisol profiles could indicate low local sensitivity and consequently low parameter precision.

### 2.4 Enzymatic Activity Model Evaluation

All key models were evaluated based on parameter plausibility and precision, goodness of fit (GOF) plots, and compared based on the difference in OFV (dOFV). Additionally, the parameters precision was evaluated using sampling importance resampling (SIR).

### 2.5 Original Model Refinement

Model refinement steps were performed including only the adult CAH data as the original model was developed using only adult data. Model refinement was performed by reinvestigating the assumption made in the development of the original model that cortisol could fully suppress ACTH secretion (I_max_ fixed to 99.9%). To this aim, a two‐step procedure was employed: First, I_max_ was fixed to progressively lower values (from 99.5% to 96.0% in steps of 0.5%) while re‐estimating parameters of the original model using healthy adult data only, and second, using the refined model parameters to perform estimation of enzymatic activity using adult CAH data.

Once a satisfactory adult model was established, the data from untreated pediatric CAH patients were included in the analysis and modeled using the same procedure described for the adult data (Figure 1, middle). Lastly, the impact of age on patients' enzymatic activity and cortisol clearance was evaluated using linear, hockey‐stick, power and exponential relationships and as a categorical covariate.

### 2.6 Simulations: Individual 24 h Patients Profiles

To simulate 24 h‐ACTH and cortisol profiles for each untreated patient from the dataset, the estimated individual enzymatic activity and individual parameters were extracted and used in the input dataset for simulations. To perform stochastic simulations (*n* = 1000), random‐effects were accounted for by sampling from the individual parameter estimates uncertainty distribution. Simulated profiles were overlapped with individual observed ACTH and cortisol concentration to visualize the model predictive performance and the extent of variability given by individual parameters uncertainty.

### 2.7 Simulations: Proof of Concept for Therapy Individualization

The potential to use the model for therapy individualization was explored by simulating the 24 h‐ACTH and cortisol profiles of the adult SW patient (body weight = 111 kg, age = 33.5 years) after administration of 15, 20, and 25 mg/day divided in 3 doses (2:1:1 ratio) of immediate‐release hydrocortisone starting at 05:00 every 4 h (q4h) and every 6 h (q6h). The resulting ACTH and cortisol profiles and the respective area under the curve (AUC) values were compared to the simulated untreated SW patient profiles and AUC values, and to a simulated healthy individual (same body weight as SW patient) ACTH and cortisol profiles and AUC values.

### 2.8 Software

PsN [29] (Perl Speaks NONMEM) v4.8.1 was used to access NONMEM v7.4.3 through Pirana v2.9.6 to perform modeling and simulation activities, while data management, visualization, and processing were performed using R v4.2.1 with RStudio v2022.07.2 + 576.

## 3 Results

### 3.1 Untreated CAH Patient Data

Data from 51 untreated CAH patients were available for the analysis. In total, 62 ACTH concentrations and 66 cortisol concentrations were available as some patients underwent multiple visits. All ACTH samples were analyzed using CLIA, and all cortisol samples except one were analyzed using CLIA. The remaining one cortisol sample was analyzed using LC–MS/MS. The dataset included data from 31 untreated adult CAH patients (from 19.0 to 55.5 years old): 1 SW patient featuring In2G variant, 3 SV patients featuring either NULL, I77T, or I172N variants, 15 NC patients featuring either P30L or V281L variants, and 12 NC‐cryptic (NCC, identified by family genetic studies) patients featuring either P453S or V281L variants. Additionally, the dataset included data from 20 pediatric CAH patients (from 2.60 to 17.3 years old): 19 NC patients featuring either R124C, P30L, or V281L variants, and 1 NCC patient featuring P482S variant.

### 3.2 Modeling of Enzymatic Activity Based on Original Model

**FIGURE 2:**
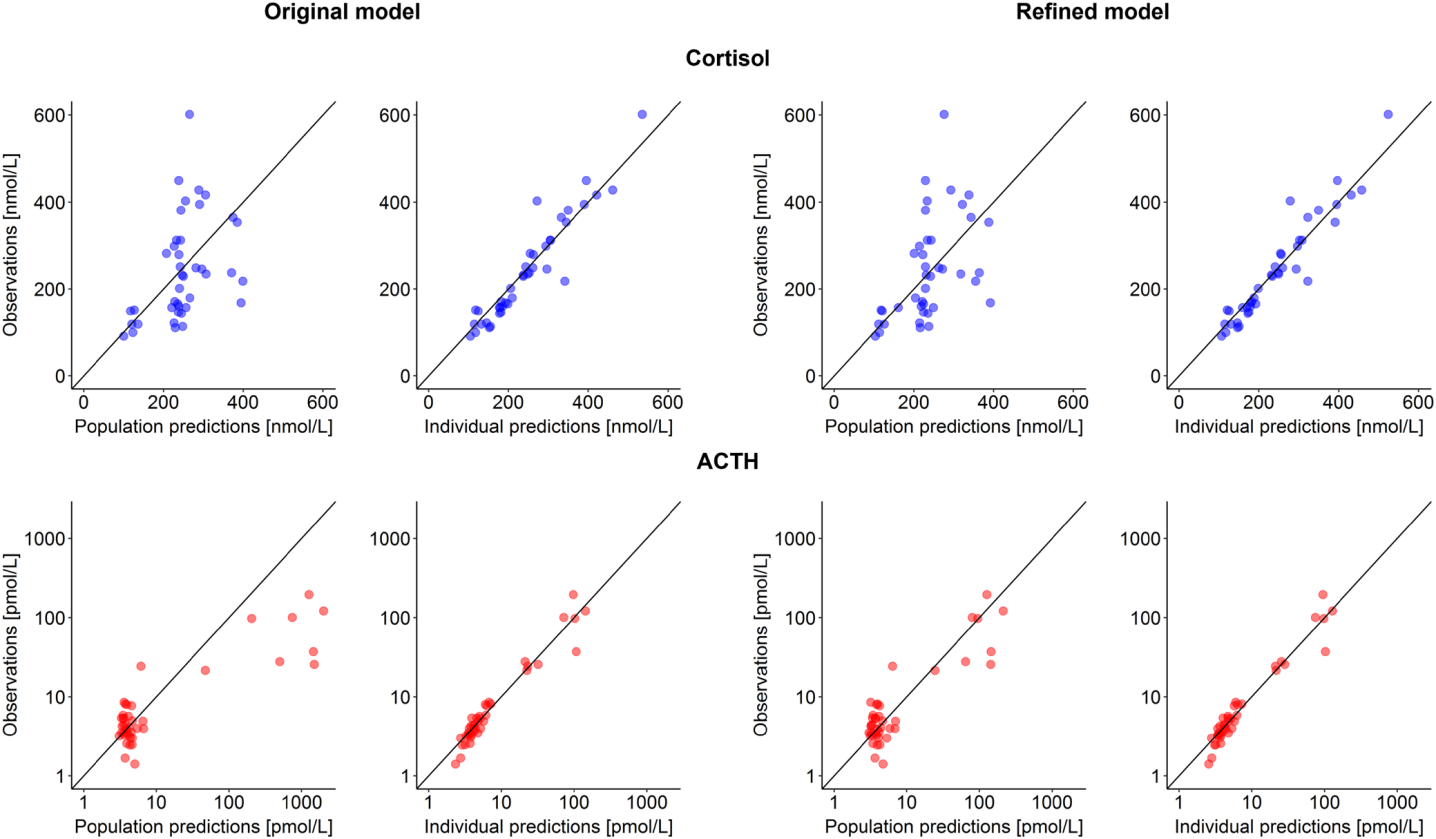
Goodness of fit plots for original model (left panels) and refined model (right panels) for cortisol concentrations on linear scale (upper panels, blue) and adrenocorticotropic hormone (ACTH) concentrations on log–log scale (bottom panels, red). Black solid line: Line of identity.

The use of the original model resulted in satisfactory ACTH and cortisol individual predictions. However, a large overprediction of high ACTH concentrations on the population level was observed, up to 10‐fold (Figure 2, bottom left: Original model). Thus, model refinement was deemed necessary.

### 3.3 Original Model Refinement

The original model parameters were re‐estimated based only on healthy adult data while fixing I_max_ (maximum cortisol‐driven ACTH suppression) to progressively lower values (99.5%, 99.0%, 98.5%, 98.0%, 97.5%, 97.0%, 96.5%, and 96.0%): This resulted in increasingly worse model performance for the healthy adult data with a dOFV of +21.9, +27.9, +33.2, +38.7, +44.5, +50.4, +56.3, and 62.2, respectively. However, when the re‐estimated parameter sets were used to estimate enzymatic activity and predict adult patient data, increasingly better model performance was observed with a dOFV of −22.2, −32.2, −36.9, −39.4, −41.5, −43.3, −43.8, and −44.3, respectively. Based on the differences between worsening model fit for healthy adults and improving model predictions for adult patients, the model with I_max_ fixed to 98% was chosen as the refined model. Importantly, the model parameters related to cortisol and hydrocortisone pharmacokinetics changed by less than 5% following model refinement, while system dynamics parameters changed: The largest impact was observed on the parameters related to the morning ACTH peak secretion, as they were intrinsically linked to I_max_. Specifically, the amplitude decreased by > 10‐fold and the width almost doubled (SA1 and SW1 in Table 1, respectively).

**TABLE 1:** Original and refined model parameter estimates.

Parameter [unit]	Parameter estimates		RSE (%)
	Original model [25]	Refined model	Refined model
ACTH secretion and elimination			
SA1 [pmol/h]	4080	357	24.6
SW1 [h]	0.607	1.03	3.00
Pt1 [hh:mm]	06:18	05:54	0.50
SA2 [pmol/h]	51.3	54.3	14.7
SW2 [h]	2.31	2.38	4.50
Pt2 [hh:mm]	11:48	12:00	0.70
*n*	4^a^	4^a^	—
K_out_ [1/h]	0.606	0.753	17.3
Base [pmol/L]	1.30	1.41	8.60
ACTH‐dependent cortisol production			
EC_50_ [pmol/L]	6.94	5.49	10.2
E_max_ [nmol/h]	5650	4010	10.2
γE	2.9	3.3	7.00
Cortisol‐dependent ACTH suppression			
IC_50_ [nmol/L]	4.58	4.51	5.30
I_max_ (%)	99.9^a^	98.0^a^	—
γI	5.35	7.09	9.00
Cortisol/hydrocortisone pharmacokinetics			
CL [L/h] (70 kg)^c^	106	108	5.10
V_C_ [L] (70 kg)^c^	2.15	2.22	7.70
Q [L/h] (70 kg)^c^	90.3	93.9	9.80
V_p_ [L] (70 kg)^c^	61.8	63.2	6.50
NS	4.15^a^	4.15^a^	—
K_d_ [nmol/L]	9.71^a^	9.71^a^	—
Hydrocortisone absorption			
F	0.344	0.350	7.50
K_a_ [1/h]	24.0	24.0^a^	—
MTT [h] (5 mg dose)	0.868	0.865	4.30
Power dose‐MTT^b^	0.179	0.179	15.8
N_tr_	2.12	2.15	10.0
Interindividual variability, CV (%)			
ω SA1	105	94.3	20.2
ω K_out_	53.4	57.8	20.7
ω Base	24.7	24.4	20.8
ω EC_50_	27.6	27.3	20.8
Covariance Base‐EC_50_	0.0626	0.0618	—
ω CL	11.4	11.6	19.2
ω V_p_	12.2	12.3	20.0
ω F	48.0	49.0	15.0
ω N_tr_	43.0	42.5	19.9
Interoccasion variability, CV (%)			
ω MTT	28.6	28.7	9.40
Residual variability, CV (%)			
*σ* ACTH_prop_	53.4	54.4	4.30
*σ* Cortisol_prop_	39.6	39.6	1.60

Abbreviations: γE, hill factor for cortisol production; γI, hill factor for ACTH suppression; ACTH, adrenocorticotropic hormone; Base, ACTH baseline concentration; B_max_, maximum binding capacity of CBG; CL, clearance; EC_50_, ACTH concentration yielding half‐maximum cortisol production; E_max_, maximum cortisol production rate constant; F, scaling factor of amount in depot; IC_50_, unbound cortisol concentration yielding half‐maximum ACTH suppression; I_max_, maximum ACTH suppression by unbound cortisol; K_a_, absorption rate constant; K_d_, dissociation constant cortisol‐CBG; K_out,ACTH_, ACTH elimination rate constant; MTT, mean transit time of oral hydrocortisone; n, surge functions exponent; NS, nonspecific binding cortisol‐albumin; N_tr_, number of transit compartments for oral hydrocortisone absorption; Pt1, peak time morning surge; Pt2, peak time midday surge; Q, intercompartmental flow; SA1, amplitude morning surge; SA2, amplitude midday surge; SW1, width morning surge; SW2, width midday surge; V_c_, central volume of distribution; V_p_, peripheral volume of distribution.

^a^ Fixed parameters.

^b^ Implemented as power covariate model.

^c^ Theory‐based allometric scaling (exponent = 0.75 for flows and = 1 for volumes).

### 3.4 Modeling of Enzymatic Activity Based on Refined Model

The predictive performance of the refined model on ACTH concentrations on the population level was largely improved compared to the original model: By visual inspection of GOF plots, no trends were observable for the refined model (Figure 2, right).

**FIGURE 3:**
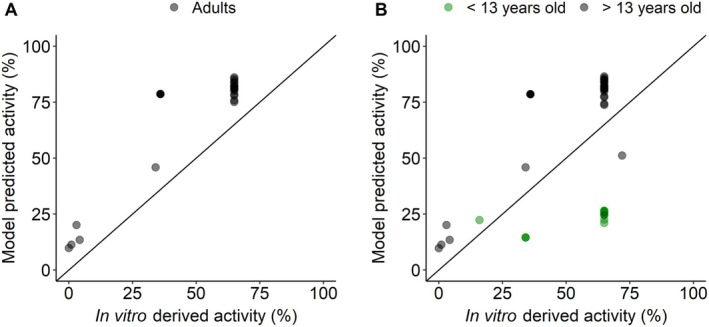
Model‐predicted individual enzymatic activity versus in vitro derived enzymatic activity (literature values). (A) Prior the inclusion of pediatric data, (B) Following the inclusion of pediatric data. Black dots: Adults, green dots: Pediatrics, black solid line: Line of identity.

The obtained enzymatic activity estimates (E_max_ scaling parameter) for adult patients were then compared to enzymatic activity values obtained from the literature, mostly quantified in in vitro studies in which the impaired 17‐hydroxyprogesterone conversion activity, that is, 21‐hydroxylase enzyme activity, was compared to the wild‐type enzymatic activity. The model‐estimated enzymatic activity typical values were plausible, yet consistently higher than literature values (Figure 3A, Table 2). Specifically, the model estimated In2G variant (SW patient) enzymatic activity was 11.3% compared to a literature genotype‐expected value of 1% [30]. Similarly, for the NULL, I77T, and I172N variants (SV patients), the model predicted enzymatic activities were 9.76%, 20.1%, and 13.5% compared to genotype‐expected values of 0%, 3%, and 4.30%, respectively [12, 31, 32]. The same trend was observed for the variants associated with NC and NCC phenotypes: For P30L, P453S, and V281L, the model predicted typical enzymatic activities were 45.9%, 78.6%, and 81.7% compared to genotype‐expected values of 34.1%, 36.0%, and 65%, respectively [12, 32, 33, 34]. Ultimately, the inclusion of interindividual variability was supported only for the V281L variant (variance in logit domain = 0.646).

**TABLE 2:** Residual enzymatic activity literature in vitro‐derived values and parameter estimates (E_max_ scaling parameter) for each pathogenic variant in the dataset for the developed pediatric and adult model.

Pathogenic variant	Residual enzymatic activity		SIR RSE (%)	LLP (95% CI, %)
	Literature values (%)	Parameter estimate (%)		
NULL	0.00	9.76	12.3	—
In2G	1.00	11.3	10.7	—
I77T	3.00	20.1	24.7	—
I172N	4.30	13.5	14.5	—
R124C	16.0	69.5^a^	—	32.6‐100
P30L	34.1	45.9^a^	—	15.6‐100
P453S	36.0	78.6^a^	—	39.7‐100
V281L	65.0	81.7^a^	—	59.8‐100
P482S	72.0	50.9^a^	—	17.7‐100
AGE effect^b^ (% to age > 13)	—	31.6	5.87	—
Interindividual variability				
ω V281L	—	0.753	34.5	—

^a^ Fixed parameters.

^b^ Implemented as proportional categorical covariate and applied to all pathogenic variants.

The refined model sensitivity analysis to E_max_ (enzymatic activity) revealed that the largest changes in ACTH and cortisol profiles were observed for E_max_ in the range between 0% and 30%, that is, In2G, NULL, I77T, and I172N variants, while the impact was less prominent in the range from 40% to 90%, that is, P30L, P453S, and V281L (Figure S2). Thus, the model sensitivity to E_max_ highlighted the lower identifiability of higher enzymatic activity estimates, negatively impacting the precision of such estimates. Additionally, the LLP for P30L, P453S, and V281L enzymatic activity parameters showed for all three parameters that a lower confidence interval limit was identifiable, while the upper confidence interval limit was not (Figure S3, Table 2). Consequently, P30L, P453S, and V281L enzymatic activity estimates were fixed to values that resulted in OFV minima from the LLP analyses.

Once a satisfactory model was established for adult patients, data from untreated pediatric CAH patients were included in the analysis. The V281L mutation was the only one well represented in both the pediatric and adult available patient data and was hence used to graphically evaluate differences in enzymatic activity between adults and children. Despite the use of only V281L for the graphical evaluation, the age effect on enzymatic activity was applied to all variants. The exploration of ETA plots in function of age showed trends for cortisol clearance and for V281L enzymatic activity (Figure S4). The inclusion of age as a categorical covariate on enzymatic activity was evaluated using different cutoff values (12, 13, 15, 18 years old and estimated cutoff), with the 13 years old cutoff resulting in the largest reduction in OFV (dOFV = −36.3): The covariate effect was modeled as a proportional scaling factor, with the enzymatic activity for patients below 13 years old estimated to be 31.6% of that in patients above 13 years old (Figure 3B). Consequently, the typical enzymatic activities for patients below 13 years old featuring P30L or V281L were 14.5% and 25.8%, respectively, compared to 45.9% and 81.7% estimated in adult patients: Additionally, the estimated enzymatic activities for patients below 13 years old were lower than in vitro derived values. Furthermore, the identified cutoff at 13 years showed that within the pediatric population, data from adolescents were more similar to adults rather than children. The inclusion of pediatrics data initially increased IIV variance on V281L enzymatic activity from 0.646 to 1.01: Following the covariate relationship implementation, IIV variance on logit domain was reduced to 0.753 (Table 2). As such, the estimated typical value and IIV variance on the logit domain corresponded to an approximate 95% range of 45.0%–96.0% for the enzymatic activity of adult patients featuring the V281L variant.

### 3.5 Simulations: Individual 24 h Patients Profiles

**FIGURE 4:**
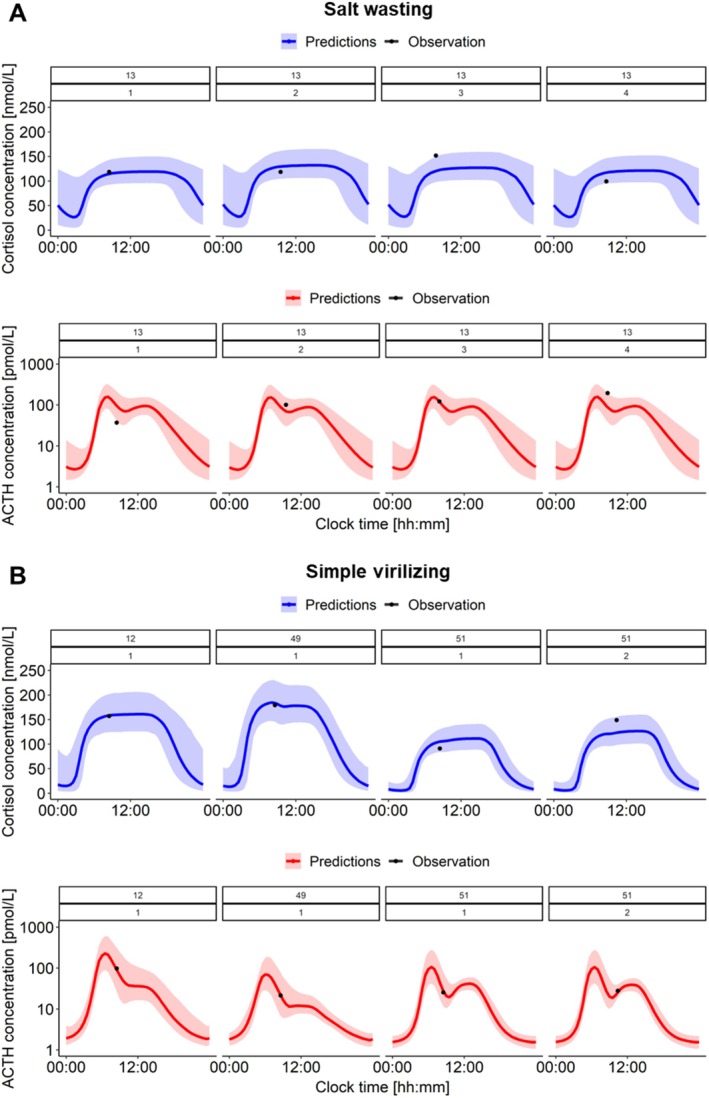
Simulations (*n* = 1000) of cortisol (blue) and adrenocorticotropic hormone (ACTH, red) 24 h concentration‐time profiles for individual untreated patients of (A) Salt wasting patient, (B) Simple virilizing patients. Colored solid lines: Median simulated profiles, colored shaded areas: 90% confidence interval of simulated profiles, black dots: Observations.

Individual ACTH and cortisol 24 h profiles were simulated (*n* = 1000) for each patient leveraging individual parameters and their uncertainty. In accordance with GOF plots (Figure 2, right: Refined model), individual observed ACTH and cortisol concentrations were well captured by the model. Most importantly, observed ACTH and cortisol concentrations always fell within, or very close to, the 90% confidence interval of simulated profiles (Figures 4, S5, S6). Additionally, the confidence intervals of simulated profiles were remarkably narrow given that only one ACTH and cortisol paired observation per patient (per occasion) was available.

### 3.6 Simulations: Proof of Concept for Therapy Individualization

**FIGURE 5:**
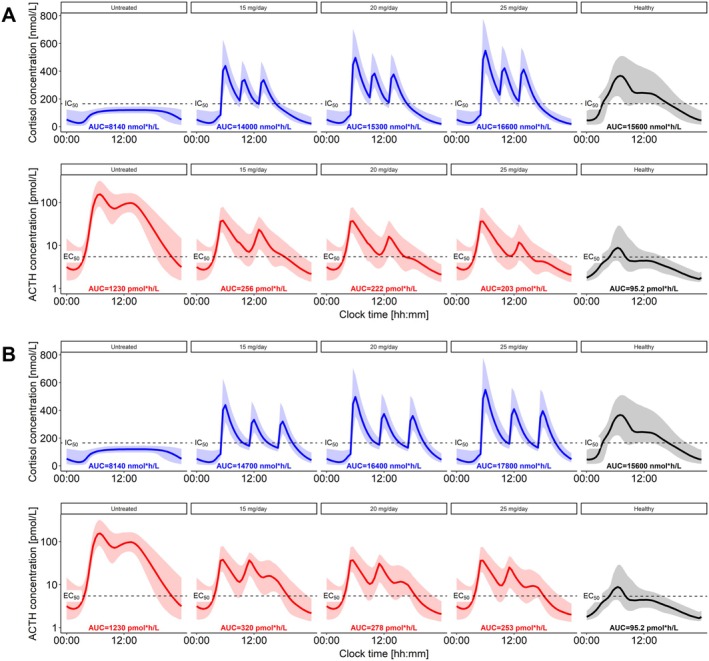
Simulations (*n* = 1000) of cortisol and adrenocorticotropic hormone (ACTH) 24 h concentration‐time profiles for untreated salt wasting patient and with hydrocortisone administration, (A) q4h, (B) q6h. Untreated (first column), 15, 20, and 25 mg/day (second, third and fourth columns, respectively). Fifth column: Simulated (*n* = 1000) healthy reference profiles. Solid lines: Median simulated profiles, shaded areas: 90% confidence interval of simulated profiles. AUC, area under concentration‐time profile; EC_50_, ACTH concentration yielding half maximum cortisol production; IC_50_, cortisol concentration yielding half maximum suppression of ACTH secretion.

Based on the simulated profiles, the untreated adult SW patient (Figure 5, left) revealed a > 10‐times higher ACTH AUC compared to a healthy individual (Figure 5, right). In this context, the therapeutic goal was to decrease the ACTH overexposure observed in the simulated untreated patient while avoiding overexposure to cortisol. In both scenarios, q4h and q6h, a trend of decreasing ACTH AUC was observed with increasing hydrocortisone daily dose (Figure 5). Additionally, the q4h dosing regimens achieved lower ACTH AUC values while maintaining lower cortisol AUC values compared to the same daily doses in the q6h dosing regimens, an important factor to avoid cortisol overexposure, and were therefore favorable. Moreover, 15 mg/day q4h achieved the same ACTH AUC reduction and lower cortisol AUC compared to 25 mg/day q6h, highlighting again the importance of dosing time. All of the administered daily doses (q4h) reduced ACTH AUC observed in the untreated patient by at least 79.1% (15 mg/day), while increasing the daily dose to 25 mg/day reduced ACTH AUC by 83.4%. For all three daily doses, cortisol AUC values were not deviating largely from the one observed in the simulated healthy individual. Despite the large improvement compared to the untreated state, none of the evaluated daily doses managed to achieve ACTH AUC values lower than double the simulated healthy individual AUC.

## 4 Discussion

In this study, we showed the potential of NLME‐based approaches to support steps towards cortisol replacement therapy individualization based on pediatric and adult CAH patient data. In particular, by refining a previously developed healthy adult NLME model characterizing ACTH and cortisol dynamics, it was possible to obtain individual enzymatic activity estimates and to predict ACTH and cortisol concentrations in untreated pediatric and adult CAH patients. Ultimately, the obtained individual enzymatic activities and parameters were leveraged to showcase the possibility of evaluating different dosing regimens for an adult patient with the most severe SW phenotype.

The need for model refinement arose based on the observation of a large overprediction of high ACTH concentration in SW and SV patients on the population level. As the original model [25] was developed using only data from healthy adults, quantitative information on ACTH overproduction in severe CAH cases were lacking. Indeed, the original model was able to predict the ACTH overproduction observed in NC and NCC patients, for which ACTH and cortisol concentration ranges were closer to those observed in healthy adults, but not in SW and SV patients, while the refined model showed good predictive performance for all 51 patients. Thus, the analysis of untreated SW and SV patients offered the opportunity to better characterize the hormonal imbalances observed in severe CAH. In some cases, and in agreement with our model refinement approach, a decreased sensitivity of feedback inhibition is clinically observed in patients with severe CAH [35]. When more data become available, the possibility to model different extents of decreased sensitivity, that is, I_max_, of feedback inhibition based on disease severity should be investigated.

The refined ACTH and cortisol model was successfully used to predict individual enzymatic activity. Overall, and especially for severe variants, the model‐estimated typical enzymatic activities were higher than previously reported values, which were mostly based on genetics and in vitro studies [13]. The source of such discrepancy could be attributed to different factors: In the presented modeling framework, the E_max_ parameter was assumed to represent 21‐hydroxylase enzyme activity, while in reality it encapsulated all processes and reactions contributing to cortisol production following adrenal stimulation by ACTH. Additionally, while in vitro studies purely measure enzymatic activity, the E_max_ parameter also accounts for enzymatic expression. The impaired enzymatic activity of CAH patients could be partly compensated by increased enzymatic expression or other unknown compensatory (non‐)HPA axis mechanisms, especially in patients who are nonadherent and no longer taking glucocorticoid medication, hence leading to higher model‐estimated enzymatic activity (E_max_). As such, the model‐based estimates can provide a more accurate patient‐specific representation compared to in vitro methodologies. Currently, genotype is often used to guide prediction of disease severity and is limited as lack of genotype–phenotype correlation commonly occurs [16, 36, 37, 38]: Moreover, genetic methodology has improved over the years but still has limitations, and incomplete or erroneous genotyping might account for some of the observed discrepancies [39]. Our study, which estimates 21‐hydroxylase deficiency based on in vivo hormone levels more accurately reflects the clinical status.

In contrast to the estimated enzymatic activities in adults, the enzymatic activity estimates for children below 13 years old were lower than previously reported values [13]. This result could hint at maturation processes of 21‐hydroxylase enzyme expression; however, to our knowledge, no data is available supporting this concept. Alternatively, it is possible that the identified relationship is a surrogate for other processes that were not accounted for, that is, differences in cortisol pharmacokinetics not captured by allometric scaling [40, 41] or differences in HPA axis activity not captured by the underlying adult ACTH‐cortisol model. Lastly, the majority of pediatric data was from NC patients. In many cases, the presentation of clinical symptoms and detection of NC CAH occurs later in life than childhood. The earlier CAH detection for the children in this analysis could represent an increased disease severity compared to typical NC patients, thus explaining the lower enzymatic activity estimates. Consequently, the extrapolation of these results to SW or SV children to evaluate dosing regimen individualization would first require further data and analyses of a larger sample size to understand whether the observed reduction in children's enzymatic activity is age‐ and/or disease severity‐related.

While showing promising results, it is essential to acknowledge some limitations of this study. The model refinement step was mostly driven by ACTH and cortisol concentrations from a small sample of SW and SV patients: In total, 8 paired ACTH and cortisol concentrations from 4 patients were available. However, untreated patients with severe CAH are a unique subpopulation of a rare disease providing valuable in vivo data for a proof‐of‐concept study. Additionally, it is rare for severe CAH patients to survive untreated [42]; therefore, possible additional systemic alterations occurring in these patients allowing them to survive were not accounted for. Whether these data can be representative of typical SW and SV patients shall be further evaluated when more data become available. Furthermore, the presented framework does not account for disease progression: Whether CAH patients undergo physiological changes that alter system dynamics and regulation cannot be characterized with the currently available data. Based on these limitations, a compromise between describing healthy and patient data was aimed for in the model refinement step: The model with I_max_ = 98% was chosen, despite not having the lowest OFV for patient data, as it was sufficient to resolve the ACTH overprediction displayed by the original model. Moreover, the potential impact of other ACTH‐driven adrenal steroids was not accounted for. Yet, while information about disease progression is lacking, the model was able to capture well all observations from the SW patient (Figure 4A): Importantly, for this patient, 13 years passed between the first and last study visit.

The lack of variability in ACTH secretion peak times, and therefore cortisol production, poses a limitation to the evaluation of individualized dosing times: All patient data analyzed in this study were assumed to follow the typical trajectory and chronotype. However, an improved therapeutic outcome was observed when shortening dosing intervals compared to increasing daily doses (Figure 5), strengthening the need to quantify and integrate time‐related variability in the framework. To quantify the variability in ACTH and cortisol peak times, in the future a larger and richer amount of paired ACTH and cortisol samples could be collected from healthy adults at least during the morning hours. Optimal design and simulation studies could help identify best sampling strategies to identify a patient's chronotype, thus allowing recommendation of individualized dosing times.

To summarize, the ability to estimate individual residual enzymatic activities in pediatric and adult patients with CAH represents a novel key step and opportunity towards individualized cortisol replacement therapy. Additionally, our study provides a potentially useful tool to evaluate disease severity beyond genotyping: The model‐based enzymatic activity estimates highlighted how genotyping and in vitro derived enzymatic activity might fail to capture patients´ clinical status. Children younger than 13 years were found to have lower enzymatic activity compared to adults: Whether this finding is age‐ and/or disease severity‐related must be further elucidated. Our analyses did not aim to recommend dosing regimens for specific patients but rather highlight the opportunity for such modeling tools to be further developed and in the future assist physicians in the decision‐making process of individualized dosing regimens. In our study, the first steps in terms of individualizing dose amount were achieved, while further studies are needed to achieve individualization of dosing times. Ultimately, this novel model‐based approach is a first step towards individualizing cortisol replacement therapy based on in vivo hormonal data.

## Author Contributions

D.B., R.M., Y.M., D.P.M., C.K., Q.L., C.S., and W.H. wrote the manuscript. D.B., R.M., D.P.M., C.K., Q.L., and C.S. designed the research. D.B. performed the research. D.B. analyzed the data.

## Conflicts of Interest

D.B., R.M., Q.L., and C.S. declare no conflicts of interest. C.K. and W.H. report grants from an industry consortium (AbbVie Deutschland GmbH and Co. K.G., Astra Zeneca, Boehringer Ingelheim Pharma GmbH & Co. K.G., F. Hoffmann‐La Roche Ltd., Merck KGaA, Novo Nordisk, and Sanofi) for the PharMetrX PhD program.

## Supporting information


Data S1.
