# Supplementary material for: Predicting Residual 21‐Hydroxylase Enzymatic Activity in Pediatric and Adult Congenital Adrenal Hyperplasia Patients: Towards Individualized Therapy
Source: CPT Pharmacometrics Syst Pharmacol. 2025 Jul 27;14(11):1869–81. doi: 10.1002/psp4.70086 (PMC12625145; doi:10.1002/psp4.70086)
Supplement: Supplementary file 1 — Data S1. [file PSP4-14-1869-s001.docx]

**Tables**

Table S1 Summary of patients characteristics and disease information

|  | **Pediatrics** | **Adults** |
| --- | --- | --- |
| Number of patients | 20 | 31 |
| *Patients characteristics, median (range)* | | |
| Age (years) | 9.45 (2.60-17.3) | 31.0 (19.0-55.5) |
| Bodyweight (kg) | 40.1 (12.8-70.0) | 84.2 (52.2-121) |
| Sex (male / female) | 8 / 12 | 7 / 24 |
| *Phenotype classification, N* | | |
| Salt wasting | 0 | 1 |
| Simple virilizing | 0 | 3 |
| Non-classic | 19 | 15 |
| Non-classic cryptic | 1 | 12 |
| *Phenotype defining pathogenic variant, N* | | |
| NULL | 0 | 1 |
| In2G | 0 | 1 |
| I77T | 0 | 1 |
| I172N | 0 | 1 |
| R124C | 1 | 0 |
| P30L | 3 | 1 |
| P453S | 0 | 5 |
| V281L | 15 | 21 |
| P482S | 1 | 0 |


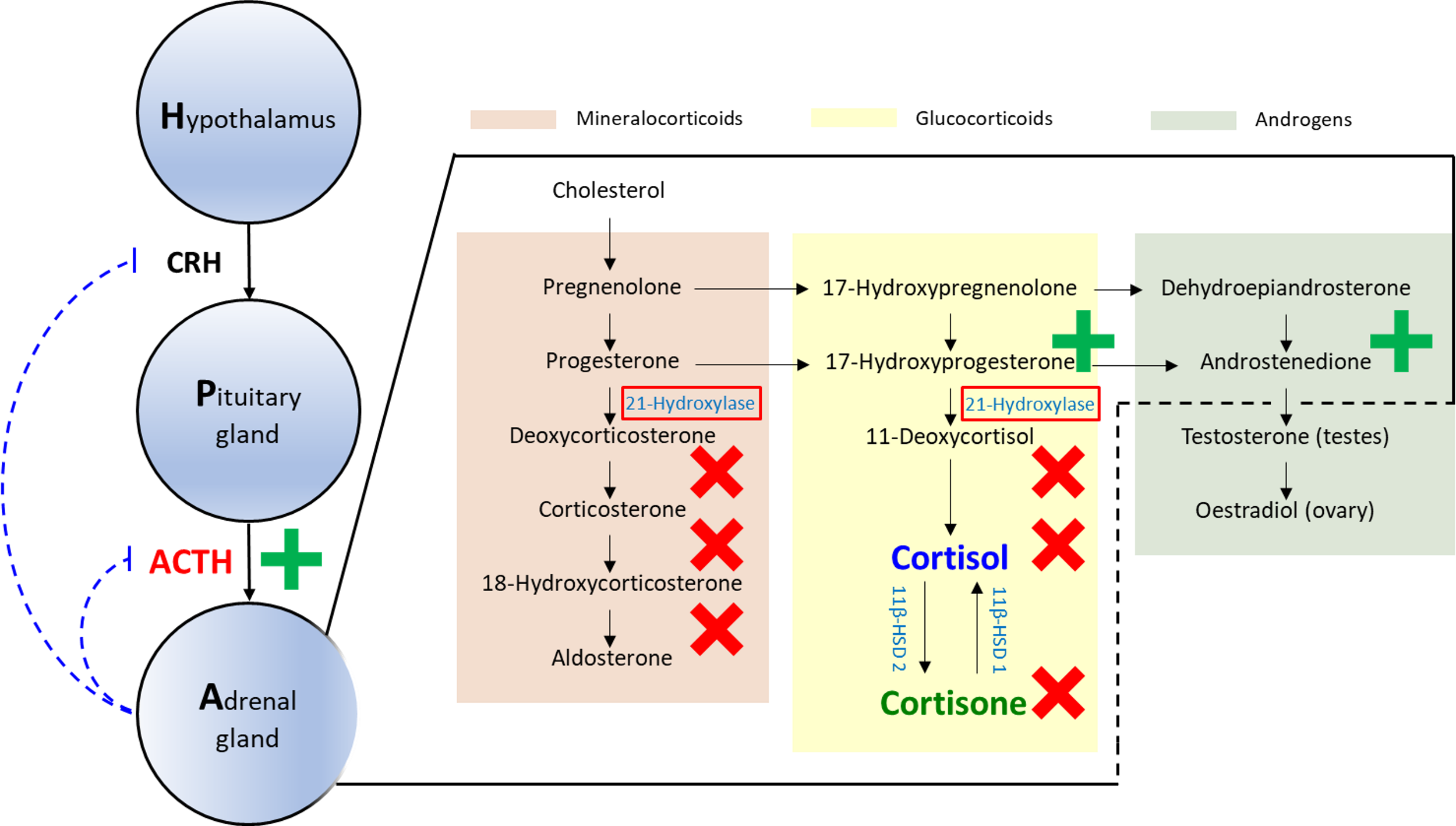
**Figures**

Figure S1 Hypothalamic-pituitary-adrenal axis and cortisol related pathways (red, green and yellow areas: Mineralocorticoid, androgens and glucocorticoid pathways, respectively) and their alterations in congenital adrenal hyperplasia with red box: deficient enzyme, red crosses: species with impaired production, green plus: overproduced species. (Adapted from Bindellini *et al.* 2024, JPKPD^26^)

ACTH: Adrenocorticotropic hormone, CRH: Corticotropic releasing hormone, 11β-HSD 1: 11β dehydrogenase type 1, 11β-HSD 2: 11β dehydrogenase type 2


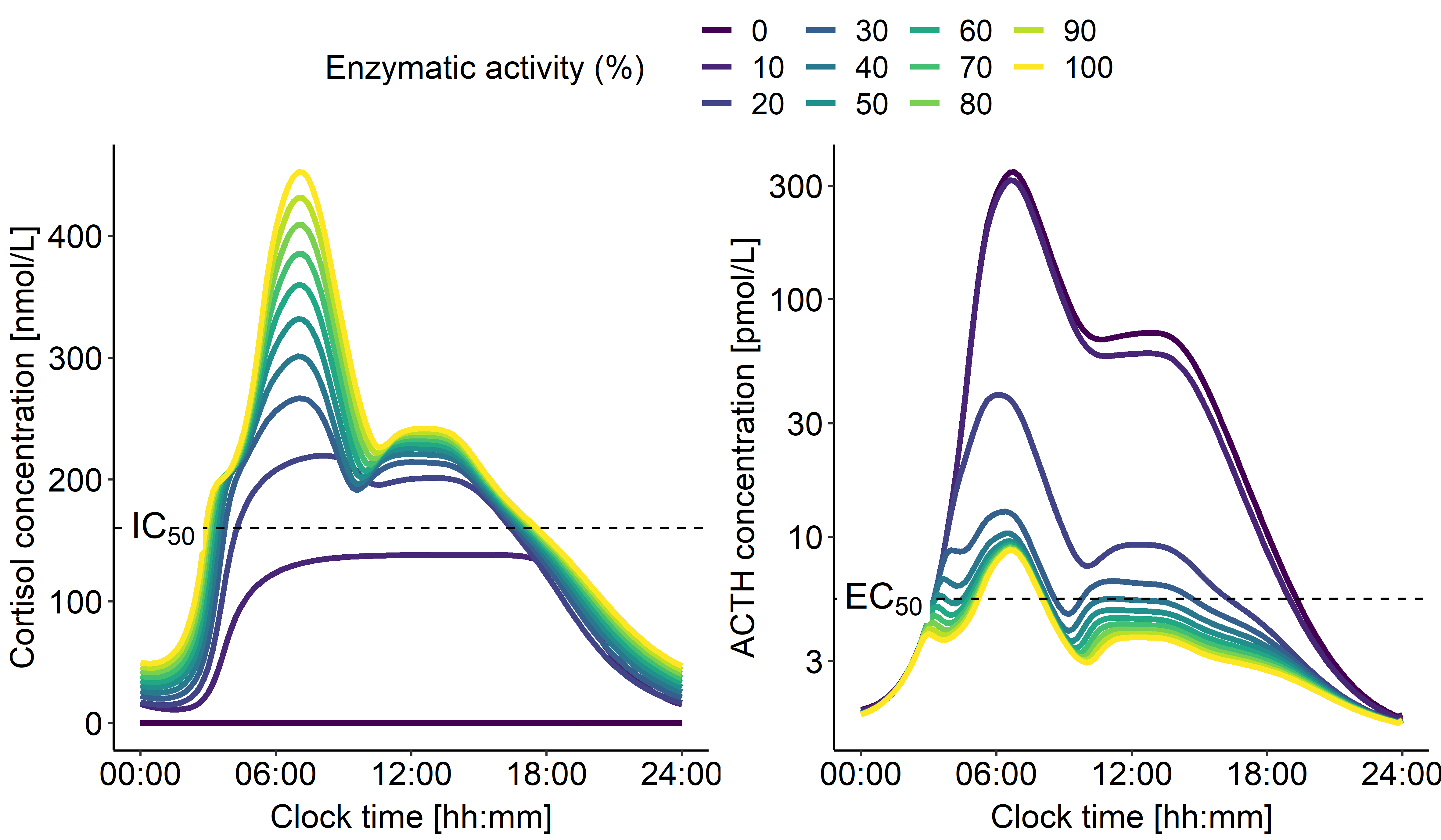


Figure S2 Cortisol (left panel) and ACTH (right panel) sensitivity to different enzymatic activity values. Colored lines: Simulated cortisol and ACTH profiles.

ACTH: Adrenocorticotropic hormone, EC50: ACTH concentration yielding half-maximum cortisol production, IC50: Unbound cortisol concentration yielding half-maximum ACTH suppression.


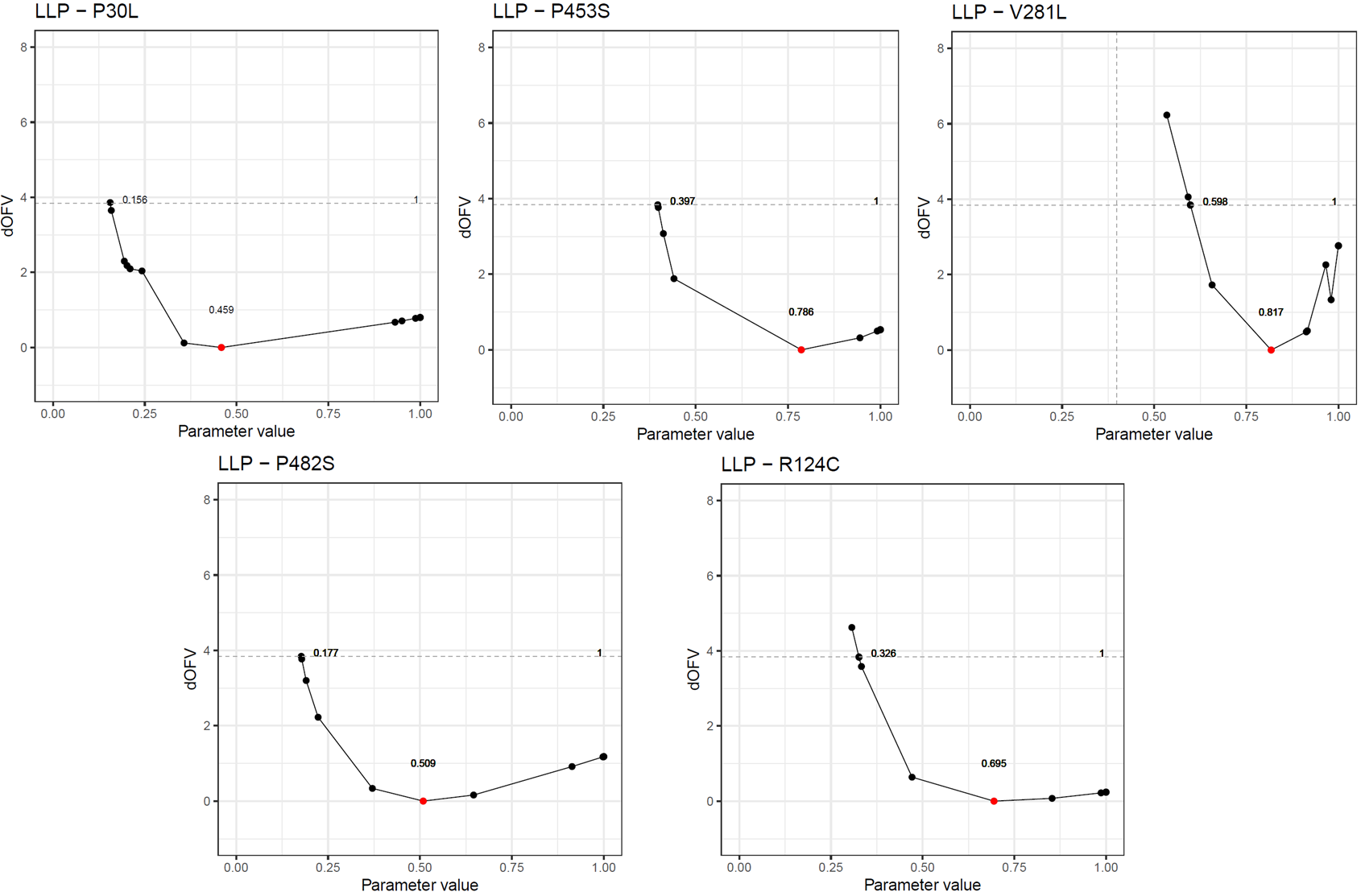


Figure S3 Log-likelihood profiling (LLP) results for different pathogenic variants. Black dots: Resulting dOFV from evaluated parameter values. Red dot: Parameter value resulting in minimum OFV.

OFV: Objective function value


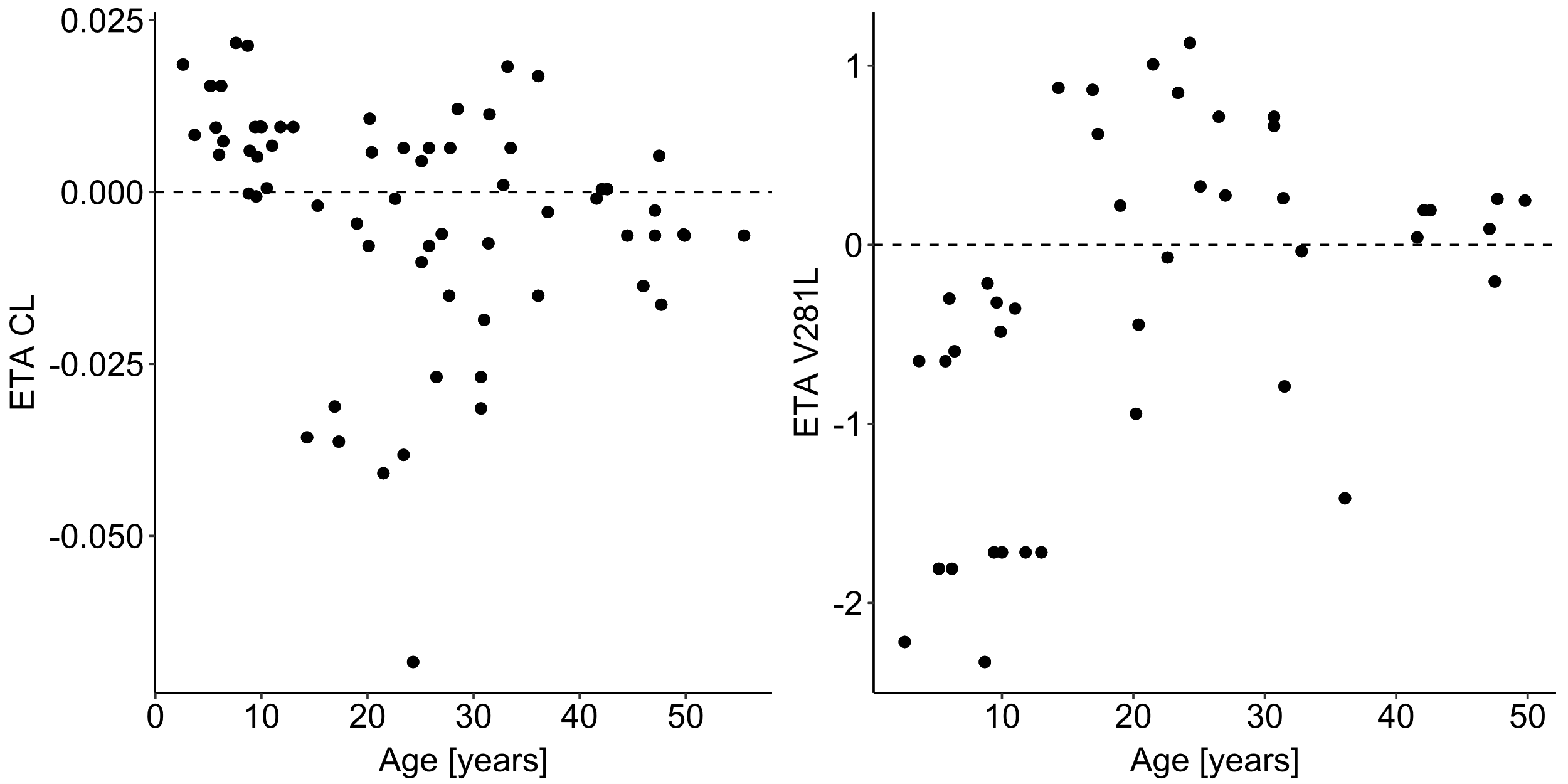


Figure S4 ETA plots for cortisol clearance (CL) and V281L variant residual enzymatic activity. Black dots: Individual ETA values, dashed line: Line y=0.


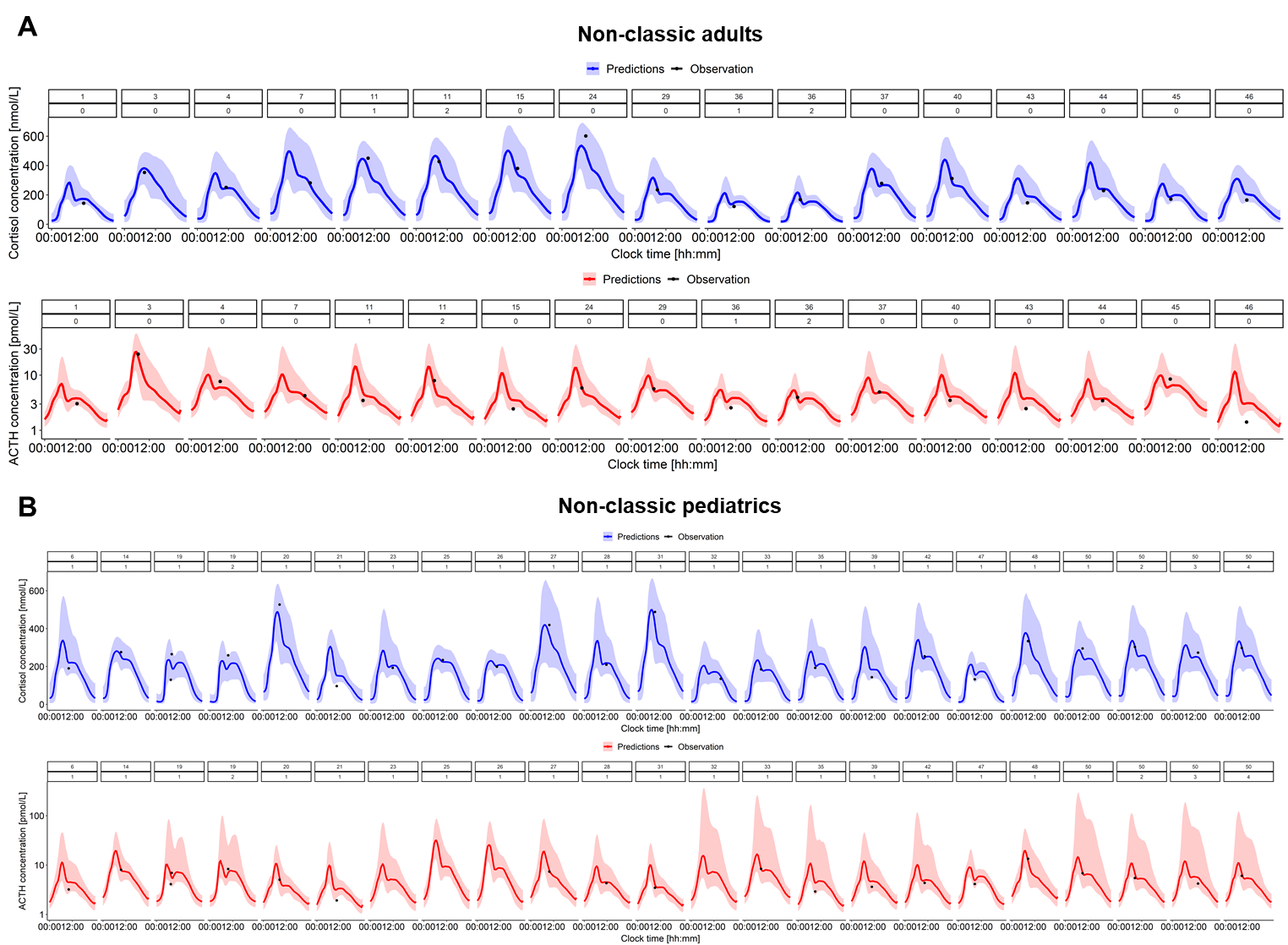


Figure S5 Simulated (n=1000) cortisol (blue) and ACTH (red) 24 hours profiles using individual parameters from NC patients. Top panels: Adult patients, bottom panels: Pediatric patients. Colored lines: Median simulated profiles, colored areas: 90% confidence interval.


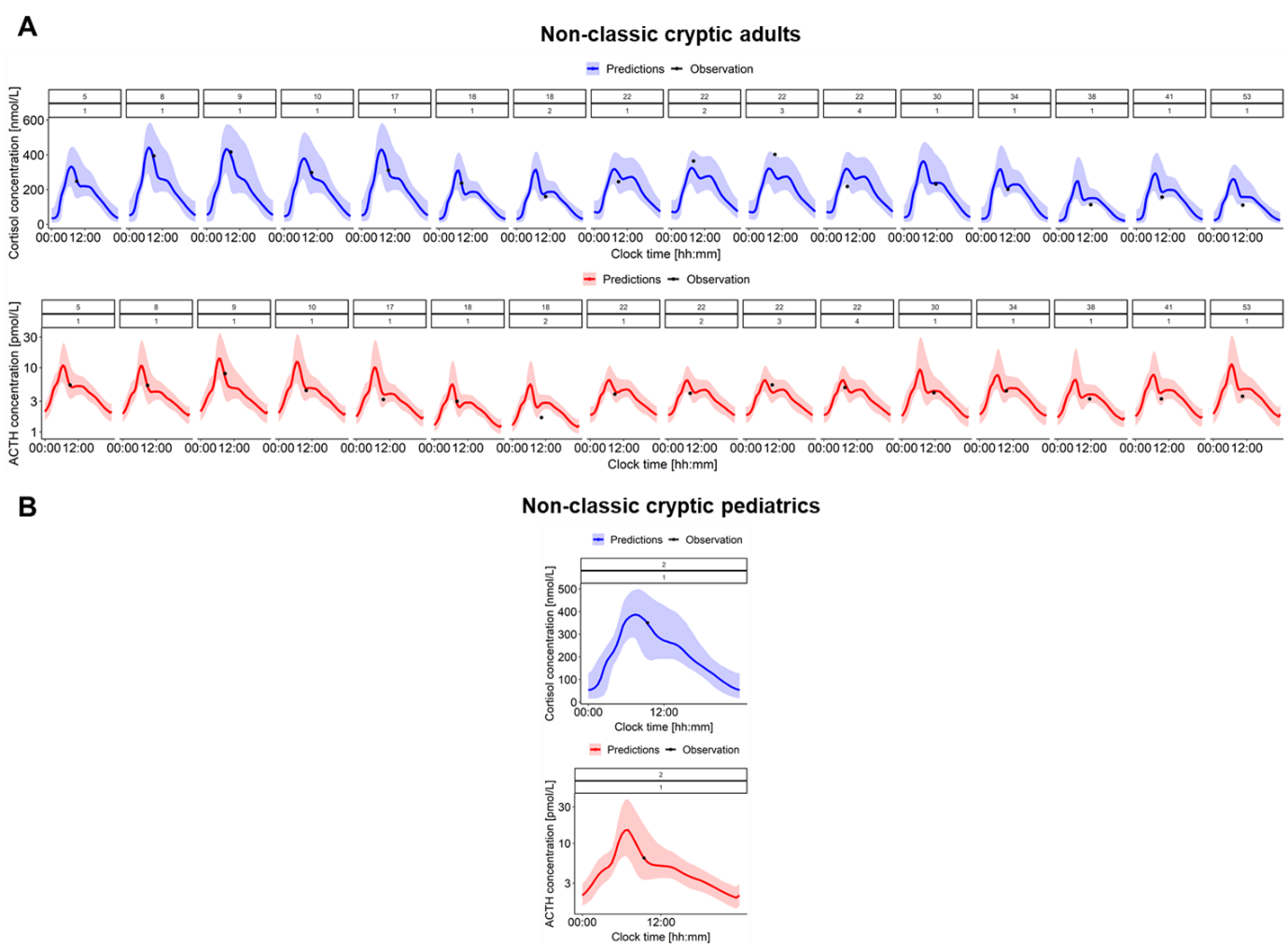


Figure S6 Simulated (n=1000) cortisol (blue) and ACTH (red) 24 hours profiles using individual parameters from NCC patients. Top panels: Adult patients, bottom panels: Pediatric patients. Colored lines: Median simulated profiles, colored areas: 90% confidence interval.

**Model equations:**

| $S1\left( t \right)=\frac{SA1}{\left( \frac{t-Pt1}{SW1} \right)^{n}+1}$  $S2\left( t \right)=\frac{SA2}{\left( \frac{t-Pt2}{SW2} \right)^{n}+1}$ | Eq. S1  Eq. S2 |
| --- | --- |
| $k_{in}=Base\cdot k_{out}$ | Eq. S3 |
| $E_{ACTH}=\frac{E_{max}\cdot{C_{ACTH}}^{\gamma E}}{{{EC}_{50}}^{\gamma E}+{C_{ACTH}}^{\gamma E}}$ | Eq. S4 |
| $FL=\frac{I_{max}\cdot{C_{u}}^{\gamma I}}{{{IC}_{50}}^{\gamma I}+{C_{u}}^{\gamma I}}$ | Eq. S5 |

| $A_{u}=\frac{A_{c}-K\cdot\left( 1+NS \right)-A_{max}+\sqrt{\left( A_{c}-K\cdot\left( 1+NS \right)-A_{max} \right)^{2}+4\cdot K\cdot\left( 1+NS \right)\cdot A_{c}}}{2\cdot\left( 1+NS \right)}$ | Eq. S6 |
| --- | --- |
| $\frac{dA_{ACTH}}{dt}=K_{in}+\left( \left( S1\left( t \right)+S2\left( t \right) \right)\cdot FL \right)-K_{out}\cdot A_{ACTH}$ | Eq. S7 |
| $\frac{dA_{depot}}{dt}=F\cdot DOSE\cdot\frac{\left( K_{tr}\cdot t \right)^{N_{tr}}}{N_{tr}!}\cdot e^{{-K}_{tr}\cdot t}-K_{a}\cdot A_{depot}$ | Eq. S8 |

| $\frac{dA_{c}}{dt}=E_{ACTH}+K_{a}\cdot A_{depot}+A_{p}\cdot\frac{Q}{V_{p}}-A_{u}\cdot\left( \frac{Q}{V_{c}}+\frac{CL}{V_{c}} \right)$ | Eq. S9 |
| --- | --- |
| $\frac{dA_{p}}{dt}={A_{u}\cdot\frac{Q}{V_{c}}-A}_{p}\cdot\frac{Q}{V_{p}}$ | Eq. S10 |

All parameter explanations are available in the footnotes of Table 1, with A_c_ and C_c_ representing the amount and concentration of total cortisol in the central compartment, A_u_ and C_u_ representing the amount and concentration of unbound cortisol, respectively, and A_p_ representing the amount of cortisol in the peripheral compartment.

**Model codes for estimation and simulation:**

<https://github.com/Kloft-Lab/Bindellini-et-al.-Untreated_CAH_Model_NONMEM_Scripts>
